# Radiation Oncology Residency Training in Saudi Arabia: Direction in Attaining Competencies

**DOI:** 10.7759/cureus.73754

**Published:** 2024-11-15

**Authors:** Amna Mohaimeed, Yasir Alayed, Saif Aljabab, Bedoor Julaidan, Mohammed Aldehaim, Firas Almomen, Abdullah Alswilem, Abdullah AlSuhaibani

**Affiliations:** 1 Department of Medicine, King Saud University Medical City, Riyadh, SAU; 2 Radiation Oncology Unit, College of Medicine, King Saud University, Riyadh, SAU; 3 Oncology Center, King Saud University Medical City, Riyadh, SAU; 4 Department of Radiation Oncology, King Faisal Specialist Hospital and Research Centre, Riyadh, SAU; 5 College of Medicine, King Faisal University, Riyadh, SAU; 6 Department of Oncology, King Saud University Medical City, Riyadh, SAU

**Keywords:** deficiencies, radiation oncology, residency program, saudi arabia, training

## Abstract

Introduction

Radiotherapy is a cornerstone in cancer treatment as a part of multidisciplinary team management. The field has evolved significantly over the past two decades due to technological advancement. To meet the increased need for manpower in the field, the radiation oncology residency program was first established in Saudi Arabia back in 2019.

Purpose

We sought to survey the trainees’ current experience across the country to establish a baseline for the different training aspects and to tackle any challenges that might go unnoticed.

Materials and methods

This is a cross-sectional survey-based study that targeted all residents enrolled in the Saudi radiation oncology training program, except the first-year residents. The trainees received an anonymous online questionnaire aiming to assess their involvement in every aspect of the daily workflow.

Results

A total of 13 eligible radiation oncology residents in Saudi Arabia were included. Eleven residents completed the survey with a national response rate of 85%. Nine (82%) of the trainees reported that they review the contours and the treatment plans with their attendings in person and this method of review is the preferred one over the hybrid method as it provides better teaching quality, interactivity, and prompt feedback. Most respondents do not review offline (7, 64%) or online (6, 55%) cone beam computed tomography (CBCT) scans with their attendings, citing the limited involvement in the approval process.

Conclusion

This study uncovered a need for better resident integration into day-to-day clinical workflow, particularly in CBCT review and approval. Addressing the current concerns will enhance the educational experience and future practice. Acknowledging the limitations of our study, we observed that certain deficiencies in core competencies reported in long-established North American programs align with our findings. This underscores the need for addressing these gaps through continuous and unbiased evaluations of radiation oncology residency training programs to enhance the educational experience and ensure high-quality preparation for independent practice.

## Introduction

Radiotherapy is a cornerstone in cancer treatment as a part of multidisciplinary team management, with more than half of all cancer patients receiving radiotherapy as part of their management [1]. The field has evolved significantly over the past two decades, partly due to technological advancement and a better understanding of radiobiology.

Radiation oncology is a highly specialized medical discipline. Training and education requirements vary across different healthcare systems. Additionally, curriculum and program structure can differ substantially depending on the local expertise and available resources [2].

The Saudi radiation oncology residency training program is a five-year training program, which was first established back in 2019 by the Saudi Commission for Health Specialties (SCFHS). This proactive measure was implemented in direct response to projections of a forthcoming increase in the number of radiotherapy centers across the Kingdom, the growing increase in population size, and the rising cancer burden due to the older age demographics. Therefore, there is a recognized future demand for competent radiation oncologists. The program structure and content were adapted from the North American curricula following the Canadian Medical Education Directives for Specialists (CanMEDS) competency model, to ensure the future demand for competent radiation oncologists can be met [3].

The present study aims to identify deficiencies and thus work on these areas for improvement, and tackle challenges in training that might go unnoticed. Additionally, it aids in building benchmarks for continuous evaluation, while encouraging us and other programs to self-examine, striving to reach well-structured training.

## Materials and methods

This is a cross-sectional online survey-based study that evaluates the perspective of Saudi radiation oncology residents regarding various clinical aspects of their training program. Thirteen radiation oncology residents from seven radiation oncology centers across the Kingdom in their 2nd to 4th year of training were included. No resident was in their final year when this study was conducted. First-year residents were excluded due to their limited exposure to radiation oncology rotations as per the program design. The questionnaire employed a multiple-choice format, including cone beam computed tomography (CBCT) approval and overall integration of trainees in the workflow and examining other factors such as respondents’ current year of training, typically used teaching modalities, preferred teaching format, and the rationale behind those preferences (Table 1).

**Table 1:** Survey questions. PGY: post-graduate year; CBCT: cone beam computed tomography.

Question	N = 11 (%)	
Which level of training are you currently completing?	PGY-2	4 (36%)
	PGY-3	5 (46%)
	PGY-4	2 (18%)
How do you review contours and plans with your attendings?	In-person	9 (82%)
	Hybrid	2 (18%)
	Virtual	0 (0%)
Which medium do you prefer (in-person, virtual, hybrid)? Select the reason for your preference.	Feedback in a timely manner	6 (55%)
	Better teaching quality	9 (82%)
	Interactivity	10 (91%)
	Convenience (i.e., location flexibility)	2 (18%)
	Increased frequency of teaching	7 (64%)
How often do you review off-line cone beam CTs for your on-treatment patients with your attending?	Weekly	0 (0%)
	Biweekly	3 (27%)
	3 times per week	1 (9%)
	I do not do this on a regular basis	7 (64%)
Do you get called to review and approve online CBCTs? If no, select the reasons why. (9 respondents)	Not part of my daily tasks as PGY-2	3 (33%)
	Busy service specialists usually get called in.	3 (33%)
	I am not familiar with the review/approval process because I was never involved	4 (44%)
In general, do you think that you are involved in every aspect of workflow?	Yes	3 (27%)
	No	1 (9%)
	Sometimes depending on my attending	6 (55%)
Which part of the workflow do you think your training is most deficient in currently? (Select all applicable)	Treatment decision-making	0 (0%)
	CT simulation approval	1 (9%)
	CBCT review/approval	7 (64%)
	Toxicity management	1 (9%)
	Plan evaluation	1 (9%)
	Follow-up plans for patients	1 (9%)
On average, how many cases do you contour per week?	1-2 cases	3 (27%)
	3-5 cases	5 (46%)
	5-7 cases	3 (27%)
On average, how many plan evaluations do you do weekly?	0 plans	3 (27%)
	1-2 plans	4 (36%)
	3-5 plans	4 (36%)

Descriptive statistics were used for data analysis, including frequency and percentage.

The study was approved by the Humanities Research Ethics Board of King Saud University (reference approval number: KSU-HE-24-697). All participants were informed about the purpose of the study and electronic informed consent was obtained from them at the beginning of the survey. The study ensures that the participants’ data are confidential and are used only for research purposes.

## Results

**Figure 1:**
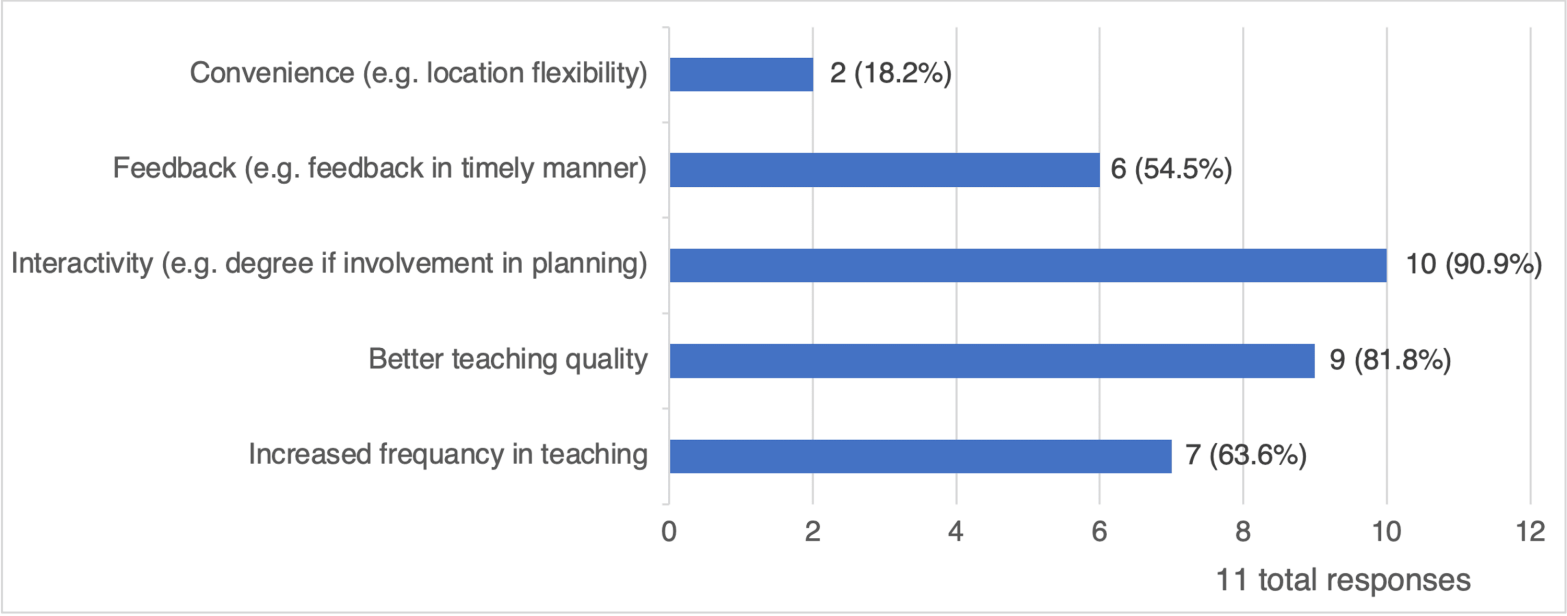
Reasons for the preferred medium of review.

With a response rate of 85%, 11 radiation oncology residents completed the survey. Only two (18%) were in their 4th year of training, five (45%) were in their 3rd year of training, and four (36%) were in their 2nd year of training. The majority (9, 82%) of the residents review the contours and the plans with their attendings in person, while only two (18%) favored the hybrid format (i.e., plans or contours are reviewed by individual consultants, then residents are provided with feedback). The preferred medium of review between the majority of residents (9, 82%) is the in-person review; they attributed the reasons behind this preference to better teaching quality, interactivity, and prompt feedback. On the other hand, only two (18%) residents preferred the hybrid method due to convenience (Figure 1).

**Figure 2:**
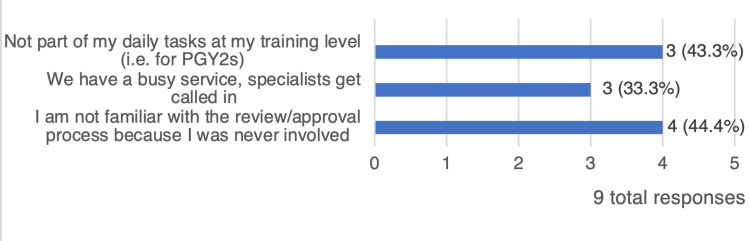
Reasons attributed to a deficiency in CBCT approvals (multiple choices). PGY2: post-graduate year 2; CBCT: cone beam computed tomography.

Most residents do not review offline and online CBCT scans with their attendings (7 (64%) and 6 (55%), respectively). This was due to various reasons: services are dependent on specialists (3, 33%), limited involvement in the approval process (4, 44%), and the review process not being part of their daily tasks as second-year residents (3, 33%) (Figure 2).

Six out of eleven residents (55%) felt that they were not involved in every part of the workflow, while only two (27%) reported their complete involvement in day-to-day workflow. Most residents (7, 70%) find themselves most deficient in CBCT review and approval.

The survey revealed that nearly half of the trainees (5, 46%) reported contouring between three and five cases per week, and the remaining trainees either contoured five to seven cases weekly (3, 27%) or one to two cases weekly (3, 27%).

Additionally, just over one-third (4, 36%) revealed that they evaluate between three and five cases per week, and another one-third (4, 36%) evaluate between one and two plans weekly. Three respondents (27%) reported a lack of plan evaluation as a part of their daily tasks, of which two were post-graduate year (PGY)-2 trainees and one was PGY-3 trainee.

## Discussion

To the best of our knowledge, this is the first study since the recent establishment of the radiation oncology residency program in Saudi Arabia that examined residents’ reported deficiencies in clinical training. The objective is to provide candid feedback to staff, mentors, program directors, and residents to help them address these identified gaps. One study revealed that 60% of radiation oncology residents based in US training programs were “not familiar” or “slightly familiar” with their competencies and after receiving their competency evaluations, 79% of those residents changed their practice and behavior [4].

Studies based on trainees’ surveys can improve program quality and foster the trainee's educational experience. A recent study that analyzed 14 US radiation oncology programs found that 34% of residents review two or fewer plans per week, with more than half of the trainees expressing deficiency in treatment plan reviews and exposure [5]. Our study revealed similar results, with 36% of residents reporting evaluating between one and two plans per week, and only 36% reporting evaluating three to five plans per week. This signifies the shared deficiencies in this area despite it being one of the core competencies [6]. Our results also revealed that 27% of residents had little to no plan review exposure during PGY-2 and PGY-3 levels, which is a concern that needs remediation and reconciliation.

Notably, the majority of the residents (70%) expressed a shortfall in CBCT review and approval, and just above half of the residents felt that they were not involved in every part of the workflow, while only two (27%) reported their complete involvement, indicating that their participation level varied based on the attending physician’s discretion.

One study conducted by the American Society for Radiation Oncology (ASTRO) reported limited involvement of residents with image-guided radiotherapy (IGRT) approvals, and only 41% of radiation oncologists involved their trainees in the online verification process. The authors emphasized the importance of training future radiation oncologists in the proper utilization of this technology [7]. Another study published by Wang et al. targeting all Canadian trainees found that half of their residents had IGRT challenges in training [8]. Our survey revealed that the only two residents who did not report any deficiencies in the CBCT review were from the same training center. These findings suggest that sharing educational and clinical training experiences may address any mutual gaps among training centers. Furthermore, these findings can support the implementation of structured CBCT seminars and mentorship.

The respondents of this study reported a preference toward in-person review of contours and plans with their attendings, and that interactivity improves their educational experience and understanding. This finding aligns with the current published data, which supports the value of such direct mentorship and feedback [9,10]. A study reported that the remote modality of contour review was implemented successfully during the COVID-19 pandemic [11]; however, this remains an unpopular approach amongst our trainees. Our results also reveal that only one resident reported a hybrid approach when reviewing contours and plans. The low preference for the hybrid method among residents is potentially attributed to it not being widely encouraged or implemented by programs [12].

Overall, radiation oncology residency programs require collaboration between physicians, physicists, and radiobiologists to cover core competency levels for residents [13,14]. Program directors (PD) especially need the resources, feedback, and dedicated time to support their critical role [15]. Effective communication between the PDs and their residents can help identify areas where the residents can be better integrated into the overall workflow. In turn, this can help PDs work on streamlining processes, and clarify roles and responsibilities of their trainees [16].

There are many limitations to our current study, one is the small sample size and the timing of the survey, as it was conducted in a newly established program, which may not have had sufficient time to mature. In addition, surveying the residents at a later stage of the program (e.g., PGY-5) may yield different results. It is important to note that due to the limited scope, caution should be exercised when generalizing these findings. Another limitation of our study is the possibility of desirability bias. This occurs when respondents provide answers they believe are more socially acceptable or favorable rather than reflecting their true thoughts, experiences, or preferences within the program. Our study mainly focused on the residents’ preferences and involvement in specific areas of training, it did not explore other aspects of the residency program such as didactic teaching, research opportunities, and overall satisfaction with training [17,18]. Addressing these limitations through a larger sample size and incorporating both subjective and objective measures and collecting longitudinal data would help strengthen the validity of these results.

## Conclusions

The launch of the Saudi radiation oncology residency program in 2019 marked an important milestone in developing cancer care expertise in Saudi Arabia. The study uncovered a need for better resident integration into day-to-day clinical workflow, particularly in CBCT review and approval. Acknowledging the limitations of our study, we observed that certain deficiencies in core competencies reported in long-established North American programs align with our findings. This underscores the need for addressing these gaps through continuous and unbiased evaluations of radiation oncology residency training programs to enhance the educational experience and ensure high-quality preparation for independent practice and foster international collaboration.
